# The Naples Prognostic Score Is Independently Associated with Paroxysmal Atrial Fibrillation: A Retrospective Case–Control Study

**DOI:** 10.3390/jcdd13080367

**Published:** 2026-08-04

**Authors:** Direnç Yılmaz, Yusuf Can, Ersin Yıldırım, Emre Eynel, Şevval Özdemir

**Affiliations:** 1 Department of Cardiology, Sakarya University Training and Research Hospital, Adapazari 54100, Turkey; direnc.yilmaz@saglik.gov.tr (D.Y.); emre.eynel@saglik.gov.tr (E.E.); sevvalozdemir@sakarya.edu.tr (Ş.Ö.); 2 Department of Cardiology, Faculty of Medicine, Sakarya University, Adapazari 54188, Turkey; 3 Department of Cardiology, Sancaktepe Şehit Prof. Dr. İlhan Varank Training and Research Hospital, Istanbul 34885, Turkey; ersin.yildirim4@saglik.gov.tr

**Keywords:** atrial fibrillation, paroxysmal, Naples prognostic score, inflammation, malnutrition, case–control studies

## Abstract

**Background and objectives:** Paroxysmal atrial fibrillation (PAF) is a common arrhythmia associated with adverse cardiovascular outcomes. Inflammation and nutritional imbalance contribute to arrhythmogenesis. The Naples Prognostic Score (NPS), derived from inflammatory and nutritional laboratory markers, has shown prognostic value in cardiovascular disease, but its association with PAF in outpatients has not been evaluated. This study assessed whether NPS is independently associated with the presence of PAF in a cardiology outpatient population. **Methods:** This retrospective case–control study included 429 patients evaluated between January 2023 and January 2025. Among them, 155 had documented PAF and 274 patients in sinus rhythm served as controls. Demographic, laboratory, and echocardiographic data were collected. NPS was calculated using serum albumin, total cholesterol, neutrophil-to-lymphocyte ratio, and lymphocyte-to-monocyte ratio. Multivariable logistic regression identified variables independently associated with PAF. **Results:** Among patients with calculable NPS (*n* = 386), NPS was higher in the PAF group (1.92 ± 0.98 vs. 0.96 ± 0.93; *p* < 0.001), and high NPS was more frequent (26.0% vs. 7.8%; *p* < 0.001). In multivariable analysis, NPS remained independently associated with documented prevalent PAF (OR 2.46; 95% CI 1.79–3.36; *p* < 0.001). **Conclusions:** NPS was independently associated with documented prevalent PAF in this outpatient cohort. These findings do not establish diagnostic or predictive utility, and prospective longitudinal studies are required to determine whether NPS is associated with incident PAF.

## 1. Introduction

Atrial fibrillation (AF) is the most common sustained cardiac arrhythmia and an increasing global public health burden [1]. It affects more than 33 million people worldwide, and its incidence is expected to rise further with population aging [2]. Approximately 1 in 4 middle-aged adults in Europe and North America will develop AF during their lifetime [3]. AF is associated with increased risks of stroke, heart failure, myocardial infarction, cognitive impairment, and mortality [4]. Its prevalence in the general adult population is estimated at 2–4%, rising with age, while many cases remain undiagnosed [5]. Paroxysmal atrial fibrillation (PAF), characterized by intermittent and self-terminating episodes, constitutes a substantial proportion of AF cases and may precede more sustained forms [6]. Although often asymptomatic, PAF is increasingly recognized as a contributor to cryptogenic stroke and reduced quality of life [7,8].

The Naples Prognostic Score (NPS) is a recently introduced scoring system that integrates inflammatory and nutritional biomarkers. It is calculated from four parameters: serum albumin, total cholesterol, neutrophil-to-lymphocyte ratio, and lymphocyte-to-monocyte ratio [9]. Originally developed in oncology, particularly for predicting outcomes after colorectal cancer surgery, NPS has shown better prognostic performance than individual inflammatory or nutritional markers [9,10]. By reflecting the interaction between chronic inflammation and malnutrition, NPS may capture processes involved in cardiovascular remodeling, endothelial dysfunction, and arrhythmogenesis. Higher NPS values have also been associated with more severe coronary artery disease, worse in-hospital outcomes after percutaneous coronary intervention, and increased long-term mortality after transcatheter aortic valve replacement (TAVR) [11,12].

Given that PAF represents one of the most frequently encountered arrhythmias in clinical cardiology, its associated clinical and biomarker-based factors remain an important area of investigation. Although several studies have focused on factors associated with PAF, the relationship between NPS and documented prevalent PAF has not been previously evaluated. Therefore, we aimed to assess whether NPS was independently associated with documented PAF in an outpatient cardiology population.

## 2. Materials & Methods

### 2.1. Study Design and Population

This retrospective, observational, case–control study included consecutive adult patients who presented to the cardiology outpatient clinic of a tertiary care center between January 2023 and January 2025 and met the eligibility criteria. The age range of the final study cohort was 18 to 92 years, reflecting the real-world distribution of eligible adult patients presenting to our outpatient clinic rather than a predefined range intended to represent the general adult population. All patients diagnosed with PAF during routine clinical evaluation in the cardiology outpatient clinic and meeting the inclusion criteria were consecutively enrolled in the study group. During the same period, the control group comprised consecutive eligible patients who presented to the same outpatient clinic for any clinical indication, were in sinus rhythm on 12-lead ECG at the index evaluation, and had no documented history of AF in the available medical records. Systematic ambulatory rhythm monitoring was not performed in all controls; therefore, the control group represented patients without documented PAF rather than a population in whom intermittent asymptomatic PAF had been definitively excluded. Thus, controls were neither randomly selected nor individually matched to PAF cases in the original study design. Baseline differences between the groups were addressed using multivariable adjustment and an additional propensity score-matched sensitivity analysis. For both groups, laboratory testing and echocardiographic evaluation were performed as part of the same index outpatient evaluation at which rhythm status was assessed. No laboratory or echocardiographic data from unrelated visits before or after the index evaluation were used. This approach was intended to minimize temporal variability and reduce potential bias related to the use of measurements obtained at different clinical encounters.

At the index evaluation, the diagnosis of PAF was based on objective rhythm documentation by standard 12-lead electrocardiography (ECG) and/or 24- to 72-h ambulatory Holter monitoring, depending on clinical indication. The diagnostic criteria were based on internationally accepted consensus guidelines [13]. Eligible participants were adult cardiology outpatients who met the group-specific criteria for documented PAF or sinus-rhythm controls described above. Exclusion criteria were autoimmune or active inflammatory disease, systemic corticosteroid therapy, thyroid disorders, active malignancy, hematologic disease, acute infection, pregnancy, and missing core clinical or echocardiographic data required for study eligibility. Missing individual laboratory variables not required for eligibility did not otherwise result in exclusion from the overall cohort; analyses involving those variables were performed using available cases. Recent blood transfusion was not a prespecified exclusion criterion because transfusion history was not systematically available in the retrospective medical records.

**Figure 1:**
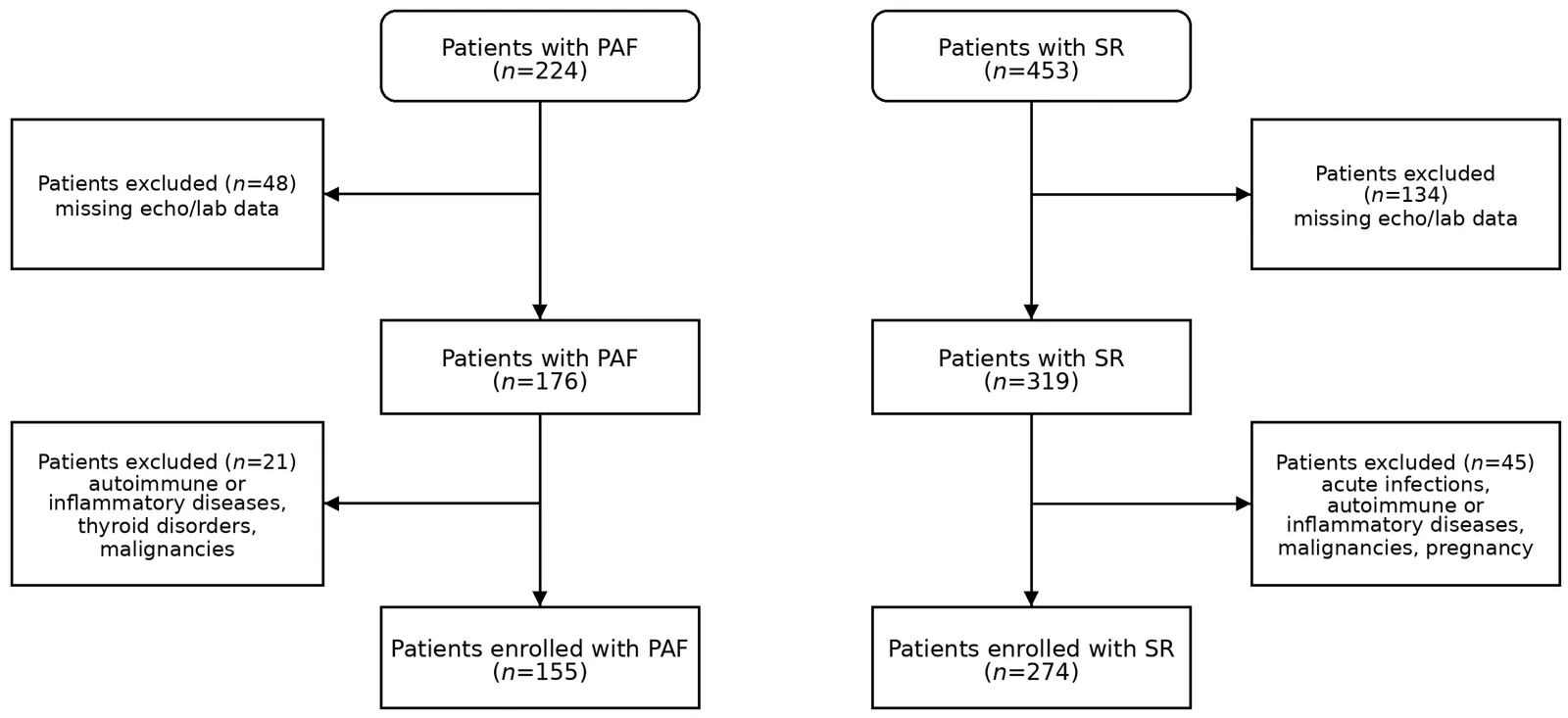
Flow diagram of patient selection, inclusion and exclusion criteria, and final analysis population.

The detailed flow diagram of patient selection and inclusion and exclusion criteria is presented in Figure 1.

### 2.2. Data Collection

Clinical and demographic characteristics, including age, sex, body mass index (BMI), and comorbidities such as hypertension, diabetes mellitus, and cerebrovascular disease, were recorded. Cerebrovascular disease was defined as a documented history of ischemic stroke or transient ischemic attack based on the medical records. Laboratory parameters were obtained from hospital records at the time of presentation and included fasting blood glucose, renal function tests (blood urea nitrogen [BUN], creatinine, and estimated glomerular filtration rate), electrolytes, serum albumin, aspartate aminotransferase (AST), and C-reactive protein (CRP). Complete blood count-derived indices including lymphocyte, monocyte, and neutrophil counts were also recorded. The lipid profile (total cholesterol, Low-Density Lipoprotein Cholesterol [LDL-C], High-Density Lipoprotein Cholesterol [HDL-C], triglycerides) was evaluated as part of routine biochemical assessment.

In addition, all patients underwent standard transthoracic echocardiographic evaluation. Left atrial diameter was measured in the parasternal long-axis view at end-systole, and left ventricular ejection fraction (LVEF) was assessed using the modified Simpson’s method, in accordance with current guideline recommendations.

### 2.3. Naples Prognostic Score Calculation

NPS is computed by assigning one point for each of the following criteria: serum albumin <40 g/L, total cholesterol ≤180 mg/dL, neutrophil-to-lymphocyte ratio >2.96, and lymphocyte-to-monocyte ratio ≤4.44. NPS was calculated only when data for all four components were available. Each parameter reflects distinct aspects of systemic inflammation or nutritional status. The total score, ranging from 0 to 4, provides a composite index that integrates metabolic and immunologic imbalance. In the primary regression analyses, NPS was entered as a continuous score. In addition, in line with prior studies, patients were stratified into low-NPS (0–2) and high-NPS (3–4) groups for descriptive purposes and to facilitate clinical interpretation [9].

### 2.4. Statistical Analysis

Statistical analyses were performed using IBM SPSS Statistics for Windows, version 23.0 (IBM Corp., Armonk, NY, USA). The distribution of continuous variables was assessed using the Kolmogorov–Smirnov and Shapiro–Wilk tests. Normally distributed data were presented as mean ± standard deviation (SD), while non-normally distributed variables were expressed as median with interquartile range (IQR). Categorical variables were summarized as frequencies and percentages. For variables with missing data, analyses were performed using available cases, and no imputation was applied. Multivariable models included patients with complete data for all variables entered into the respective model.

Group comparisons were conducted using the independent-samples *t*-test or Mann–Whitney U test for continuous variables, and the chi-square test or Fisher’s exact test for categorical variables, as appropriate. Variables considered clinically relevant and/or statistically associated with PAF in univariable analyses were entered into the multivariable logistic regression model to identify variables independently associated with the presence of PAF. Multicollinearity among variables was assessed using variance inflation factors (VIFs). Odds ratios (ORs) with corresponding 95% confidence intervals (CIs) were reported.

To examine the relative contributions of the individual NPS components, an additional analysis was performed in patients with complete data for serum albumin, total cholesterol, NLR, and LMR. Two separate multivariable logistic regression models adjusted for age, sex, hypertension, diabetes mellitus, coronary artery disease, cerebrovascular disease, smoking status, alcohol use, body mass index, left atrial diameter, and BUN were constructed: one including NPS and the other including its four individual components. NPS and its individual components were not entered into the same model because NPS is mathematically derived from these variables. Total cholesterol was modeled per 10 mg/dL increase.

To further address baseline imbalance, an additional propensity score-matched sensitivity analysis was performed among patients with calculable NPS. Propensity scores were estimated using a logistic regression model including age, sex, hypertension, diabetes mellitus, coronary artery disease, cerebrovascular disease, smoking status, alcohol use, body mass index, and left atrial diameter. Optimal one-to-one pair matching without replacement was performed on the logit of the propensity score using a caliper of 0.2 standard deviations. Covariate balance was assessed using absolute standardized mean differences, with values below 0.10 indicating adequate balance. The association between NPS and documented PAF in the matched cohort was evaluated using conditional logistic regression. Because a small residual imbalance remained for sex after matching, an additional sex-adjusted conditional logistic regression model was also performed.

An exploratory ROC analysis was performed as a supplementary analysis and is presented in Supplementary Figure S1. A two-tailed *p*-value < 0.05 was considered statistically significant.

### 2.5. Ethical Considerations

The study protocol was conducted in accordance with the Declaration of Helsinki and was approved by the institutional ethics committee. All data were collected retrospectively from electronic hospital records, and patient confidentiality was strictly maintained throughout the study. As this was a retrospective analysis of anonymized data, the requirement for informed consent was waived.

## 3. Results

A total of 429 patients were enrolled, including 155 (36.1%) with PAF and 274 (63.9%) in sinus rhythm. Because of the case–control design, the proportion of patients with PAF in the study cohort reflects the sampling structure and should not be interpreted as an estimate of PAF prevalence among all cardiology outpatients. The mean age of the entire cohort was 58.71 ± 12.45 years, and 53.2% were female. The distribution of patients’ baseline demographic and clinical characteristics is presented in Table 1.

The gender distribution was comparable between the two groups, with no statistically significant difference observed (*p* = 0.143). Although AF is generally reported to be more prevalent in men, the sex distribution in our cohort may reflect the characteristics of our single-center outpatient population rather than the epidemiology of AF in the general population. Patients in the PAF group were significantly older compared to the control group (63.38 ± 11.83 vs. 55.46 ± 11.86 years, *p* < 0.001). The prevalence of hypertension (69.7% vs. 48.2%, *p* < 0.001) and cerebrovascular disease (16.1% vs. 1.8%, *p* < 0.001) was significantly higher in the PAF group compared to the control group, whereas no significant differences were observed in terms of diabetes mellitus, smoking status, or alcohol use (Table 1).

Abbreviations: BMI, body mass index; BUN, blood urea nitrogen; CRP, C-reactive protein; HDL-C, high-density lipoprotein cholesterol; IQR, interquartile range; LDL-C, low-density lipoprotein cholesterol; LMR, lymphocyte-to-monocyte ratio; NLR, neutrophil-to-lymphocyte ratio; NPS, Naples Prognostic Score; PAF, paroxysmal atrial fibrillation; PIV, pan-immune-inflammation value; SD, standard deviation; SII, systemic immune-inflammation index; TSH, thyroid-stimulating hormone.

Echocardiographic and laboratory assessments revealed that PAF patients had significantly larger left atrial diameters (38.35 ± 5.30 vs. 33.65 ± 4.30 mm, *p* < 0.001) and higher body mass index (28.87 ± 4.87 vs. 27.46 ± 4.44 kg/m^2^, *p* = 0.002). Among laboratory parameters, BUN and CRP levels were notably elevated in the PAF group compared to the control group (35.60 ± 12.14 vs. 29.98 ± 8.95 mg/dL, *p* < 0.001 and 14.43 ± 27.19 vs. 6.46 ± 18.63 mg/dL, *p* = 0.003, respectively). In contrast, serum albumin levels were significantly lower in patients with PAF (37.09 ± 3.50 vs. 43.25 ± 2.34 g/L, *p* < 0.001).

NPS could be calculated in 386 patients, including 255 controls and 131 patients with PAF. Among these patients, NPS was higher in the PAF group than in the control group (1.92 ± 0.98 vs. 0.96 ± 0.93; *p* < 0.001), and the high-NPS category was more frequent in the PAF group (34/131 [26.0%] vs. 20/255 [7.8%]; *p* < 0.001).

Univariable logistic regression analysis identified several variables as significantly associated with the presence of PAF, including age (OR 1.06; 95% CI 1.04–1.08; *p* < 0.001), hypertension (OR 2.47; 95% CI 1.63–3.75; *p* < 0.001), cerebrovascular disease (OR 10.35; 95% CI 3.87–27.64; *p* < 0.001), BMI (OR 1.07; 95% CI 1.02–1.11; *p* = 0.003), left atrial diameter (OR 1.23; 95% CI 1.17–1.30; *p* < 0.001), BUN (OR 1.07; 95% CI 1.04–1.09; *p* < 0.001), CRP (OR 1.02; 95% CI 1.01–1.03; *p* = 0.004), creatinine (OR 4.75; 95% CI 1.97–11.44; *p* = 0.001), and NPS (OR 2.65; 95% CI 2.07–3.40; *p* < 0.001) (Table 2).

The re-estimated multivariable model included 355 patients with complete data for all variables entered into the model. Variables independently associated with documented prevalent PAF were cerebrovascular disease (OR 6.92; 95% CI 1.57–30.47; *p* = 0.010), left atrial diameter (OR 1.19; 95% CI 1.11–1.27; *p* < 0.001), BUN (OR 1.04; 95% CI 1.01–1.08; *p* = 0.009), and NPS (OR 2.46; 95% CI 1.79–3.36; *p* < 0.001).

Among the 386 patients with calculable NPS, optimal propensity score matching yielded 93 matched PAF–control pairs. Following matching, the baseline variables specifically highlighted by the reviewer were adequately balanced, with absolute standardized mean differences of 0.028 for age, 0.067 for hypertension, 0.056 for cerebrovascular disease, 0.008 for body mass index, and 0.085 for left atrial diameter. In conditional logistic regression analysis of the matched cohort, NPS remained significantly associated with documented prevalent PAF (OR 2.44; 95% CI 1.62–3.69; *p* < 0.001). Because a small residual imbalance remained for sex after matching (absolute standardized mean difference, 0.129), an additional sex-adjusted analysis was performed, and the association remained significant (OR 2.61; 95% CI 1.69–4.05; *p* < 0.001) (Supplementary Table S1).

In the complete-component cohort (*n* = 386), serum albumin showed the most prominent component-level association with documented prevalent PAF (OR per 1 g/L increase, 0.21; 95% CI 0.13–0.32; *p* < 0.001), whereas total cholesterol, analyzed per 10 mg/dL increase (OR 1.04; 95% CI 0.94–1.14; *p* = 0.486); NLR (OR 1.00; 95% CI 0.72–1.40; *p* = 0.978); and LMR (OR 1.17; 95% CI 0.95–1.45; *p* = 0.142) were not independently associated with PAF. In a separate model adjusted for the same clinical covariates, NPS remained significantly associated with documented prevalent PAF (OR per 1-point increase, 2.78; 95% CI 2.03–3.82; *p* < 0.001) (Supplementary Table S2).

In an exploratory ROC analysis, NPS showed moderate discrimination between patients with and without documented prevalent PAF within the study cohort, with an AUC of 0.753. Detailed cut-off, sensitivity, and specificity findings are presented in Supplementary Figure S1.

## 4. Discussion

To the best of our knowledge, this is the first study to demonstrate a significant association between the Naples Prognostic Score and paroxysmal atrial fibrillation in an outpatient cardiology population. In this retrospective, case–control analysis, we found that patients with PAF had significantly higher NPS values compared to those in sinus rhythm. Moreover, multivariable regression analysis revealed that NPS was independently associated with the presence of PAF, even after adjusting for well-established clinical and echocardiographic variables. Importantly, the association between NPS and documented PAF remained significant in the propensity score-matched cohort, supporting the robustness of the main finding after improved balance of measured baseline characteristics. These findings support an association between higher NPS and documented prevalent PAF; however, the component-level analysis suggests that this association may be driven largely by serum albumin. The findings do not establish a temporal or predictive relationship.

Systemic inflammation has been increasingly recognized as a pivotal contributor to the initiation and maintenance of atrial fibrillation, including its paroxysmal form. Pro-inflammatory cytokines and immune cell activation can lead to atrial structural remodeling and electrical instability, fostering arrhythmogenesis [14]. Traditional inflammatory markers such as C-reactive protein, interleukin-6 (IL-6), and NLR have been associated with AF onset and recurrence [15,16]. In our study, patients with PAF exhibited significantly higher CRP levels compared to those in sinus rhythm, supporting the hypothesis that inflammation contributes to atrial vulnerability. Recently, novel composite inflammatory indices such as the Systemic Immune-Inflammation Index (SII) have emerged. Calculated as (platelet count × neutrophil count)/lymphocyte count, SII has shown potential in predicting AF recurrence after surgical ablation [17]. In parallel, a new prognostic biomarker, the Pan-Immune-Inflammatory Value (PIV), derived from (neutrophil × platelet × monocyte)/lymphocyte counts, has been proposed as a broader indicator of overall immune system activation, involving both early and long-term immune responses [18]. Elevated PIVs have been significantly associated with early AF recurrence following cryoablation, suggesting that multidimensional inflammatory scores may provide enhanced predictive power over single biomarkers [19].

The NPS was initially introduced in the oncologic setting to predict postoperative outcomes in patients with colorectal cancer [9]. Originally introduced in oncology, the NPS has recently garnered attention in cardiovascular medicine for its potential to reflect both inflammatory and nutritional status; two elements increasingly recognized as central to the pathophysiology of a wide spectrum of cardiac disorders. Chronic inflammation contributes to endothelial dysfunction, plaque instability, atrial remodeling, and myocardial fibrosis, while malnutrition is known to impair cardiac repair mechanisms, immune function, and overall survival [20,21]. The NPS, by integrating biomarkers such as serum albumin, total cholesterol, neutrophil-to-lymphocyte ratio, and lymphocyte-to-monocyte ratio, provides a practical and cost-efficient means of simultaneously capturing both domains.

In the component-level analysis, serum albumin showed the most prominent association with documented prevalent PAF, whereas total cholesterol, NLR, and LMR were not independently associated. This finding is broadly consistent with a previous matched case–control study in which serum albumin levels were lower in patients with PAF than in controls and an independent inverse association between albumin and PAF was demonstrated in male patients [22]. The pattern observed in our cohort suggests that the association between NPS and PAF may be driven largely by serum albumin rather than by uniform contributions from all four components. Although NPS remained associated with PAF in a separate model, these analyses do not establish that the composite score provides incremental information beyond serum albumin alone. These component-level findings should be considered exploratory and require confirmation in external cohorts.

Several studies have highlighted the prognostic utility of the NPS in various cardiac conditions. For instance, elevated NPS has been associated with increased in-hospital mortality and adverse outcomes in patients with acute ST-elevation myocardial infarction (STEMI) [23], as well as with worse long-term prognosis in heart failure populations [24]. Furthermore, higher NPS values have been linked to a greater incidence of major adverse cardiovascular events (MACE) following transcatheter aortic valve replacement [25], and to increased long-term mortality in patients with peripheral artery disease undergoing revascularization [26]. Similarly, a recent population-based study demonstrated that higher NPS values were independently associated with increased risk of incident stroke and all-cause mortality among older adults [27]. Consistent with our findings, a recent study demonstrated that elevated NPS levels were independently associated with the development of postoperative AF following isolated coronary artery bypass grafting (CABG), highlighting the potential role of the NPS in identifying patients prone to arrhythmogenesis beyond conventional risk factors [28]. In the exploratory ROC analysis, NPS showed moderate discrimination within the present cohort. However, this internally derived finding was not externally validated and should not be interpreted as evidence of screening, diagnostic, or predictive utility.

Taken together, to the best of our knowledge, this is the first study to demonstrate an independent association between NPS and documented prevalent PAF. However, given the cross-sectional design of the study, these results should not be interpreted as evidence that NPS can diagnose PAF or predict its future development. NPS cannot replace established rhythm-based diagnostic methods, including 12-lead ECG, ambulatory Holter monitoring, or wearable rhythm-monitoring devices. Prospective longitudinal studies are warranted to determine whether NPS is associated with incident PAF and to further clarify the clinical relevance of this association.

## 5. Study Limitations

Several limitations of this study should be acknowledged. First, the retrospective and single-center design, conducted in a tertiary referral hospital, may limit the generalizability of the findings to broader populations, as the patient profile may differ from community-based or primary care settings. Second, rhythm assessment was based on standard ECG and clinically indicated 24- to 72-h Holter monitoring, and monitoring intensity was not standardized between groups. Because systematic ambulatory rhythm monitoring was not performed in all controls, asymptomatic or intermittent PAF may have remained undetected in some patients classified as controls. This potential misclassification limits the certainty of the observed association, and variability in Holter monitoring duration may also have influenced diagnostic sensitivity. Accordingly, the observed association should be interpreted as an association with documented prevalent PAF rather than with PAF definitively excluded in the control group. Symptom status, time since the first documented PAF diagnosis, episode frequency and duration, and overall AF burden were not systematically available in the retrospective records and could therefore not be evaluated. Because NPS and PAF status were assessed at the same index evaluation, the study cannot establish temporal precedence or predict future PAF. The exploratory ROC analysis was internally derived and not externally validated and should therefore not be interpreted as evidence of screening, diagnostic, or predictive performance. Third, while the NPS provides valuable insight into systemic inflammation and nutritional status, it is a static measure that may vary over time and be affected by transient physiological or pathological states. Information regarding recent blood transfusion was not systematically available in the retrospective records; therefore, a potential influence of transfusion on laboratory parameters included in the NPS could not be fully excluded. NPS could not be calculated in 43 patients because one or more component values were missing; NPS-related analyses were therefore performed using available cases without imputation. In addition, AF is a multifactorial disorder, and the present study was not designed to capture other important contributors such as autonomic dysfunction or atrial structural remodeling. Furthermore, although the NPS and its cut-off values were originally derived from oncology studies and have been explored in cardiovascular settings, these thresholds are less clearly defined and less specific in cardiovascular populations compared with oncology. Although propensity score matching substantially improved the balance of measured baseline characteristics, residual confounding related to unmeasured or incompletely measured factors cannot be entirely excluded. Finally, the moderate overall sample size, particularly the propensity score-matched cohort of 93 pairs, limits the precision and generalizability of the matched findings. The absence of an external validation cohort further underscores the need for prospective, multicenter studies to confirm our results.

## 6. Conclusions

In this retrospective case–control study, to the best of our knowledge, we demonstrated for the first time that the Naples Prognostic Score was independently associated with documented prevalent PAF in an outpatient cardiology population. These findings support an association between NPS and the clinical profile of patients with prevalent PAF but do not establish screening, diagnostic, or predictive utility. Prospective longitudinal studies are warranted to validate these findings and to determine whether NPS is also associated with incident PAF.

## Figures and Tables

**Table 1:** Baseline characteristics and laboratory findings of the groups.

Variables	Sinus Rhythm (*n* = 274)	PAF (*n* = 155)	*p* Value
*Baseline characteristics*			
Age, years, mean (SD)	55.46 ± 11.86	63.38 ± 11.83	<0.001
Female sex, *n* (%)	139 (50.7)	90 (58.1)	0.143
Coronary artery disease, *n* (%)	60 (21.9)	38 (24.5)	0.535
Hypertension, *n* (%)	132 (48.2)	108 (69.7)	<0.001
Diabetes mellitus, *n* (%)	56 (20.4)	40 (25.8)	0.200
Cerebrovascular disease, *n* (%)	5 (1.8)	25 (16.1)	<0.001
Current smoking, *n* (%)	79 (28.8)	33 (21.3)	0.088
Alcohol use, *n* (%)	20 (7.3)	11 (7.1)	0.938
BMI, kg/m^2^, mean (SD)	27.46 ± 4.44	28.87 ± 4.87	0.002
Left atrial diameter, mm, mean (SD)	33.65 ± 4.30	38.35 ± 5.30	<0.001
*Laboratory findings*			
Glucose, mg/dL, mean (SD)	113.05 ± 42.91	118.35 ± 50.29	0.251
Lymphocyte count, ×10^3^/µL, mean (SD)	2.41 ± 0.95	2.24 ± 0.78	0.063
Neutrophil count, ×10^3^/µL, mean (SD)	4.81 ± 1.74	4.74 ± 1.97	0.727
Monocyte count, ×10^3^/µL, mean (SD)	0.54 ± 0.42	0.50 ± 0.24	0.302
Albumin, g/L, mean (SD)	43.25 ± 2.34	37.09 ± 3.50	<0.001
BUN, mg/dL, mean (SD)	29.98 ± 8.95	35.60 ± 12.14	<0.001
Creatinine, mg/dL, median (IQR)	0.76 (0.61–0.88)	0.81 (0.66–1.00)	0.002
Total cholesterol, mg/dL, mean (SD)	199.17 ± 51.13	193.93 ± 51.89	0.331
HDL-C, mg/dL, mean (SD)	49.60 ± 12.20	48.10 ± 11.73	0.069
LDL-C, mg/dL, mean (SD)	133.69 ± 53.91	127.10 ± 39.28	0.200
Triglycerides, mg/dL, mean (SD)	149.73 ± 87.39	157.99 ± 98.40	0.389
Hemoglobin, g/dL, mean (SD)	13.65 ± 1.58	13.94 ± 1.74	0.694
Platelet count, ×10^3^/µL, mean (SD)	270.39 ± 65.81	253.68 ± 79.37	0.020
TSH, µIU/mL, mean (SD)	1.99 ± 2.61	1.91 ± 1.70	0.779
CRP, mg/dL, mean (SD)	6.46 ± 18.63	14.43 ± 27.19	0.003
NLR, mean (SD)	2.28 ± 1.43	2.39 ± 1.63	0.463
LMR, mean (SD)	4.92 ± 1.99	5.33 ± 3.63	0.129
PIV, mean (SD)	332.22 ± 346.58	334.32 ± 436.87	0.956
SII, mean (SD)	601.10 ± 357.03	594.06 ± 388.56	0.849
NPS, mean (SD)	0.96 ± 0.93	1.92 ± 0.98	<0.001
High NPS (3–4), *n/N* (%)	20/255 (7.8)	34/131 (26.0)	<0.001

Values are presented as mean ± SD, median (IQR), or n (%), as appropriate. Group comparisons used the independent-samples *t* test, Mann–Whitney U test, chi-square test, or Fisher exact test, as appropriate. Analyses were performed using available cases without imputation. NPS was calculable in 255 patients with sinus rhythm and 131 patients with PAF.

**Table 2:** Logistic regression analyses of factors associated with documented prevalent PAF.

	Univariable Analysis		Multivariable Analysis	
Variable	OR (95% CI)	*p* Value	OR (95% CI)	*p* Value
Age, per year	1.06 (1.04–1.08)	<0.001	0.99 (0.97–1.02)	0.635
Hypertension	2.47 (1.63–3.75)	<0.001	1.55 (0.82–2.90)	0.175
Cerebrovascular disease	10.35 (3.87–27.64)	<0.001	6.92 (1.57–30.47)	0.010
BMI, per 1 kg/m^2^	1.07 (1.02–1.11)	0.003	1.05 (0.99–1.12)	0.112
Left atrial diameter, per 1 mm	1.23 (1.17–1.30)	<0.001	1.19 (1.11–1.27)	<0.001
BUN, per 1 mg/dL	1.07 (1.04–1.09)	<0.001	1.04 (1.01–1.08)	0.009
CRP, per 1 mg/dL	1.02 (1.01–1.03)	0.004	1.01 (1.00–1.02)	0.152
Creatinine, per 1 mg/dL	4.75 (1.97–11.44)	0.001	1.46 (0.82–2.59)	0.196
NPS, per 1-point increase	2.65 (2.07–3.40)	<0.001	2.46 (1.79–3.36)	<0.001

Univariable analyses were performed using available cases for each predictor. The multivariable model included 355 patients with complete data for all variables entered into the model. Abbreviations: BMI, body mass index; BUN, blood urea nitrogen; CI, confidence interval; CRP, C-reactive protein; NPS, Naples Prognostic Score; OR, odds ratio; PAF, paroxysmal atrial fibrillation.

## Data Availability

The datasets used or analyzed during the current study are available from the corresponding author on reasonable request.

## Supplementary Materials

The following supporting information can be downloaded at: https://www.mdpi.com/article/10.3390/jcdd13080367/s1, Figure S1: Exploratory receiver operating characteristic analysis of the Naples Prognostic Score for documented prevalent paroxysmal atrial fibrillation within the study cohort; Table S1: Covariate balance before and after propensity score matching and the association between the Naples Prognostic Score and documented paroxysmal atrial fibrillation in the matched cohort; Table S2: Separate multivariable models evaluating NPS and its individual components in the complete-component cohort.

## Abbreviations

AF	Atrial fibrillation
AST	Aspartate aminotransferase
AUC	Area under the ROC curve
BMI	Body mass index
BUN	Blood urea nitrogen
CABG	Coronary artery bypass grafting
CI	Confidence interval
CRP	C-reactive protein
ECG	Electrocardiogram
HDL-C	High-density lipoprotein cholesterol
IQR	Interquartile range
LDL-C	Low-density lipoprotein cholesterol
LMR	Lymphocyte-to-monocyte ratio
LVEF	Left ventricular ejection fraction
MACE	Major adverse cardiovascular events
NLR	Neutrophil-to-lymphocyte ratio
NPS	Naples Prognostic Score
OR	Odds ratio
PAF	Paroxysmal atrial fibrillation
PIV	Pan-immune-inflammation value
ROC	Receiver operating characteristic
SD	Standard deviation
SII	Systemic immune-inflammation index
STEMI	ST-elevation myocardial infarction
TAVR	Transcatheter aortic valve replacement
TSH	Thyroid stimulating hormone
VIFs	Variance inflation factors
