# Supplementary material for: The Naples Prognostic Score Is Independently Associated with Paroxysmal Atrial Fibrillation: A Retrospective Case–Control Study
Source: J Cardiovasc Dev Dis. 2026 Aug 4;13(8):367. doi: 10.3390/jcdd13080367 (PMC13513197; doi:10.3390/jcdd13080367)
Supplement: Supplementary file 1 [file jcdd-13-00367-s001.zip › jcdd-4445576-supplementary.pdf]

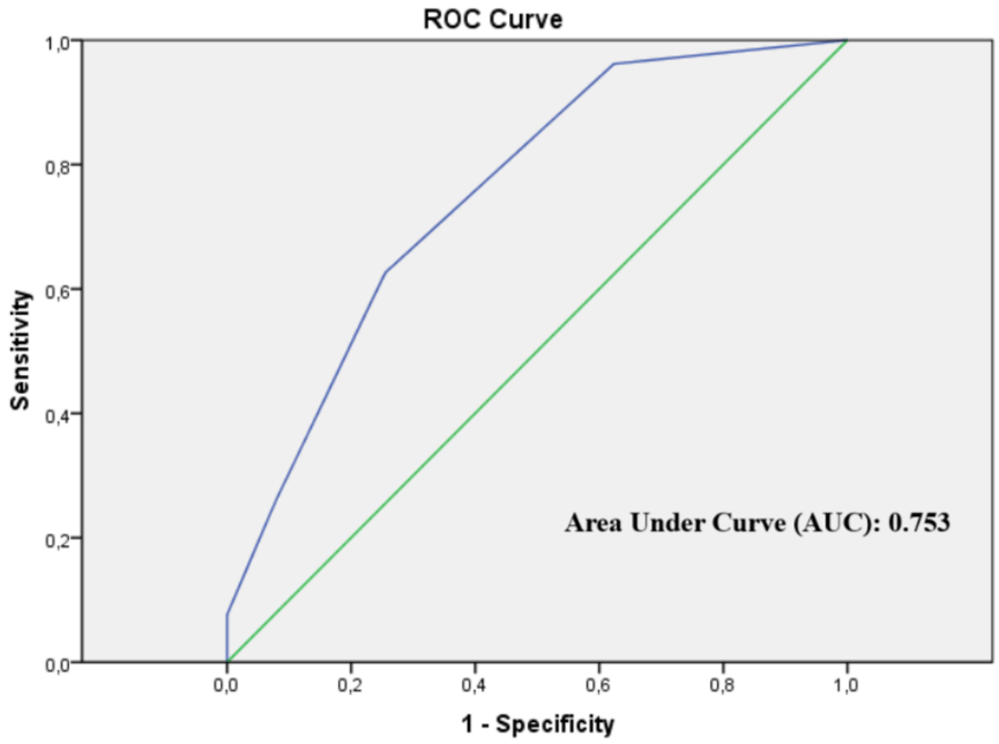

Figure S1. Exploratory receiver operating characteristic analysis of the Naples Prognostic Score for documented prevalent paroxysmal atrial fibrillation within the study cohort. The area under the curve was 0.753. The internally derived cut-off of 1.5 yielded a sensitivity of 62.6% and specificity of 74.5%. These findings were not externally validated and should not be interpreted as evidence of screening, diagnostic, or predictive performance.

Table S1. Covariate balance before and after propensity score matching and the association between the Naples Prognostic Score and documented paroxysmal atrial fibrillation in the matched cohort.

Panel A. Covariate balance before and after propensity score matching

Covariate	Absolute SMD before matching	Absolute SMD after matching
Age	0.668	0.028
Sex	0.071	0.129
Hypertension	0.406	0.067
Diabetes mellitus	0.069	0.026
Coronary artery disease	0.040	0.025
Cerebrovascular disease	0.526	0.056
Smoking status	0.124	0.076
Alcohol use	0.024	0.056
Body mass index	0.291	0.008
Left atrial diameter	0.988	0.085

Panel B. Conditional logistic regression in the matched cohort

Model	OR	95% CI	p value
Matched cohort	2.44	1.62-3.69	<0.001
Matched cohort, additionally adjusted for sex	2.61	1.69-4.05	<0.001

Propensity scores were estimated using age, sex, hypertension, diabetes mellitus, coronary artery disease, cerebrovascular disease, smoking status, alcohol use, body mass index, and left atrial diameter.
Optimal one-to-one pair matching without replacement was performed on the logit of the propensity score using a caliper of 0.2 standard deviations and yielded 93 matched PAF-control pairs (186 patients).
An absolute standardized mean difference below 0.10 was considered to indicate adequate balance. A small residual imbalance remained for sex after matching (absolute SMD, 0.129); therefore, an additional sex-adjusted model was performed.
Odds ratios represent the association with documented PAF per 1-point increase in NPS.
Abbreviations: CI, confidence interval; NPS, Naples Prognostic Score; OR, odds ratio; PAF, paroxysmal atrial fibrillation; SMD, standardized mean difference.

Table S2. Separate multivariable models evaluating NPS and its individual components in the complete-component cohort.

Predictor	Adjusted OR (95% CI)	p value
Model A: Composite NPS		
NPS, per 1-point increase	2.78 (2.03–3.82)	<0.001
Model B: Individual NPS components		
Albumin, per 1 g/L increase	0.21 (0.13–0.32)	<0.001
Total cholesterol, per 10 mg/dL increase	1.04 (0.94–1.14)	0.486
NLR	1.00 (0.72–1.40)	0.978
LMR	1.17 (0.95–1.45)	0.142

Both models included 386 patients with complete data for all four NPS components and were adjusted for age, sex, hypertension, diabetes mellitus, coronary artery disease, cerebrovascular disease, smoking status, alcohol use, body mass index, left atrial diameter, and BUN.

NPS and its individual components were evaluated in separate models because NPS is mathematically derived from albumin, total cholesterol, NLR, and LMR. No imputation was performed.

Abbreviations: BMI, body mass index; BUN, blood urea nitrogen; CI, confidence interval; LMR, lymphocyte-to-monocyte ratio; NLR, neutrophil-to-lymphocyte ratio; NPS, Naples Prognostic Score; OR, odds ratio.
